# Algebraic Topology of Multi-Brain Connectivity Networks Reveals Dissimilarity in Functional Patterns during Spoken Communications

**DOI:** 10.1371/journal.pone.0166787

**Published:** 2016-11-23

**Authors:** Bosiljka Tadić, Miroslav Andjelković, Biljana Mileva Boshkoska, Zoran Levnajić

**Affiliations:** 1 Department of Theoretical Physics, Jožef Stefan Institute, 1001 Ljubljana, Slovenia; 2 Institute for Nuclear Sciences Vinča, University of Belgrade, Belgrade, Serbia; 3 Faculty of Information Studies, Ulica Talcev 3, 8000 Novo Mesto, Slovenia; 4 Department of Knowledge Technologies, Jožef Stefan Institute, 1001 Ljubljana, Slovenia; University of North Carolina at Chapel Hill, UNITED STATES

## Abstract

Human behaviour in various circumstances mirrors the corresponding brain connectivity patterns, which are suitably represented by functional brain networks. While the objective analysis of these networks by graph theory tools deepened our understanding of brain functions, the multi-brain structures and connections underlying human social behaviour remain largely unexplored. In this study, we analyse the aggregate graph that maps coordination of EEG signals previously recorded during spoken communications in two groups of six listeners and two speakers. Applying an innovative approach based on the algebraic topology of graphs, we analyse higher-order topological complexes consisting of mutually interwoven cliques of a high order to which the identified functional connections organise. Our results reveal that the topological quantifiers provide new suitable measures for differences in the brain activity patterns and inter-brain synchronisation between speakers and listeners. Moreover, the higher topological complexity correlates with the listener’s concentration to the story, confirmed by self-rating, and closeness to the speaker’s brain activity pattern, which is measured by network-to-network distance. The connectivity structures of the frontal and parietal lobe consistently constitute distinct clusters, which extend across the listener’s group. Formally, the topology quantifiers of the multi-brain communities exceed the sum of those of the participating individuals and also reflect the listener’s rated attributes of the speaker and the narrated subject. In the broader context, the presented study exposes the relevance of higher topological structures (besides standard graph measures) for characterising functional brain networks under different stimuli.

## Introduction

In the past few years, a big leap in understanding the structure and function of the human brain has been provided with both advances in brain imaging techniques [1, 2] as well as the use of complex networks perspective to analyse the emerging empirical data [3]. Currently, active research differentiates two aspects of brain networks, representing anatomic and functional connections between distinct brain regions [4–7]. Anatomical connections are chiefly investigated by diffusion tensor imaging. The functional brain connectivity, on the other hand, can be detected at different spatial and temporal scales. In this regard, functional magnetic resonance imaging (fMRI) captures synchronisation among blood-oxygenation-level-dependent signals at a good spatial resolution and low frequency. Much shorter time scales can characterise the brain connections related to different brain function, for instance, information processing, integration or segregation, cognitive control, empathy, and other. Therefore, electroencephalography (EEG) imaging has received an increased interest in functional brain research [8–12]. In this case, the functional connections are often reconstructed from EEG signals recorded at many scalp locations. In contrast to fMRI imaging, which measures spatially specific cortical or subcortical regions, the signal registered by an electrode at a particular scalp location (i.e., above a cortical region of interest) is spatially less specific, containing the average electric neuronal activities of all voxels belonging to that area [8, 12]. Nevertheless, regarding the generalised synchronisation, the recognisable patterns of positively correlated EEG signals suitably reflect the macroscopic organisation of the brain network [3]. Thus, the underlying brain activity corresponding to a variety of situations has been analysed through EEG-based connections, for example, processing (un)pleasant music [13], the objective identification of emotions [10] or the pathological changes in the context of epilepsy [11], anesthetic agents induction [14], and other.

Brain anatomical connections are suitably represented by weighted networks. In this case, there is a growing consensus about the confidence level that a particular link is present as well as its weight [15]. On the other hand, a variety of functional connections have been observed, closely reflecting a particular brain activity, that map to a different functional network [4]. Such examples of the brain networks include the recently studied functional paths in integration and segregation of information [16], inter-regional communication [17], convergence of information in hippocampus [18], stimulus selection [19], cognitive control circuits [20], as well as the effects of different stimuli [19, 21, 22], learning [23], perception of time, numbers and languages [24, 25], the presence of a mental disease [26] and more. Although the anatomical connections lay the basis, the functional brain networks often appear to have a richer structure, which is attributed to dynamical factors: the appearance of longer paths as well as the avalanches of the cascading activity propagation. Thus, clearly distinguishing between the brain activity patterns related to particular mental processes remains a challenging task.

In the neuroscience research, a central problem is how to explain the brain function from microscopic, biochemical processes, on one side, and its impact on human behaviour, on the other. Recently, concerns on how the brain mediates social interactions, which is closely related both to cognitive and affective neuroscience, lead to the development of the social neurology. In this respect, the research of the social impact on the processes in the brain [27–29] as well as the brain processes that underly an effective social behaviour [30–38] is becoming a subject of increasing interest. New approaches using simultaneous scanning of groups of participants are being developed to study social cognition [39, 40]. The studies of face-to-face communications in dyads reveal a significant degree of synchronisation in particular brain areas, depending on the performed task [34–36, 38]. Whereas, other types of communications seem to involve different mechanisms [35]. Furthermore, a higher brain activation level characterises the leader compared to its follower, the game builder compared to its partner, as well as the same individual performing the cooperation compared to the competition role [37]. In contrast to the extensive study of functional brain connections, as stated above, little attention has been devoted [38, 40] to analyse multi-brain graphs and to identify the social impact onto the functional brain networks.

In this work, we study a complex network of brains of a group of individuals during spoken communications; we map an aggregate data of EEG signals, previously recorded in the experiment described in [41]. In the experiment, two different narrations of the speakers are *superimposed* and presented to the two groups of six listeners at the same time, while a group’s aim was to focus to a particular speaker. The EEG signals were recorded simultaneously at all listeners during the session, while the recording was performed independently for each speaker during his/her narration, see Methods. Note also that, in contrast to the platforms build on the face-to-face communications [39], in this experimental set-up the interaction is unidirectional from speakers to listeners. The self-rated experiences of the listeners collected after each completed session, indicate wide variations of the listener’s concentration to the story, correlating to the previous knowledge, interest, as well as the speaker’s attractiveness and narrative quality.

The previous study of the data [41] focused to the statistically significant group-averaged features, in particular of the speaker-listener coordination. The aim of the present analysis is entirely different. We reveal the fine structure of the aggregate multi-brain network considering the location of each EEG electrode as a network node. Thus our approach, based on mapping the correlations among EEG signals, allows us to analyse the networks of connections elicited by the presented stimulus in each listener’s brain as well as the speaker–listener and listener–listener coordination, and the emerging cross-brain structures. We hypothesised that, while involving the similar brain areas, the brain activity patterns of each participant (depending on its role in the session, cognitive and emotional content communicated by the speakers, and other factors) results in different connectivity in the corresponding functional networks and brain-to-brain connections. To analyse these significant differences between brain networks, we develop an approach based on the algebraic topology of graphs, which identifies higher-order structures containing cliques of a large order and their aggregates beyond the standard network parameters. The applied methodology offers a new perspective in the analysis of inter-brain synchronisation during social communications and other functional brain networks besides the social brain problems.

## Materials and Methods

### EEG data acquisition and preprocessing

**Fig 1:**
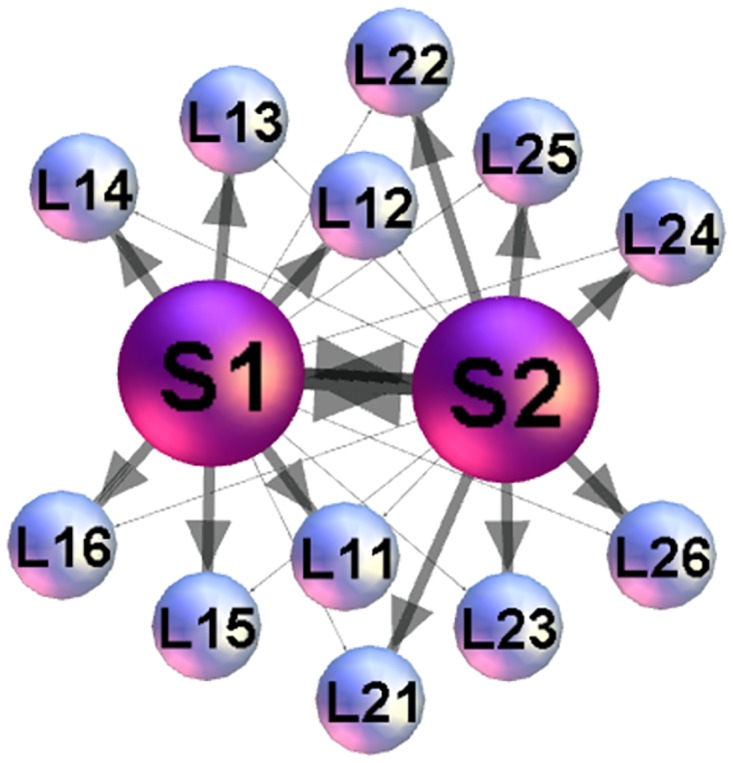
Schematic presentation of the set up with two speakers S1 and S2 and 12 listeners. The listeners *k* = 1, 2⋯6 of each group *L*_1−*k*_ and *L*_2−*k*_ are instructed to follow the narration of a particular speaker, indicated by the heavy lines, while having the narration of the other speaker simultaneously accessible, shown by the thin lines.

We use the empirical data of two sessions, each session containing a different stimulus, from the previously recorded data set of [41]. A stimulus consists of two superimposed audiovisual recordings of a similar duration (about 4 minutes) where two speakers, female and male, narrate different stories to the video cameras. As described in [41], a specific software was used to superimpose onto each other the two faces of the speakers and the soundtracks of their voices. Thus obtained video was adjusted so that both speakers appeared equally prominent. The stimulus is then presented to twelve listeners. While the input was the same to all listeners, one group of six listeners was instructed to attend to one speaker and the other group to the other speaker. The attended speaker was introduced to each panel in the first 5 seconds of the stimulus. The schematic illustration of the experimental setup is shown in Fig 1. In each stimulus, EEG recordings from two speakers are made during freely narrating stories, while the EEG scanning of all listeners is performed simultaneously during the session.

We consider data of two different stimuli, in particular,

- *Stimulus1* consists of the storytelling by a male speaker (S1) and a female speaker (S2), and the corresponding two groups of six listeners. Recalled by the speaker’s own words, the narration is based on written versions of international fairytales.
- *Stimulus11* contains an ad-hoc invented narration by a female speaker S3, attended by the listener’s group 2, and a free narration from the favourite book or movie of the speaker S1 (the same speaker as in stimulus1), attended by the group 1. For these narrations, no written text was available to the speakers.

The EEG signals were acquired from 63 scalp locations positioned according to the International 10/20 System. A detailed description of the EEG acquisition and preprocessing is given in the original paper [41]. According to [41], the preprocessing included the necessary steps in which (i) EEG recordings were aligned with the corresponding audio signal for each participant; (ii) the noise was reduced by applying 50Hz notch filter; (iii) artifactual components (i.e., due to speaking, eye movement) were removed using an advanced technique based on the independent component analysis, which can separate the signal components of different origin, and rating. Moreover, the data were transformed to the average reference and temporally trimmed to the overlapping time segments.

The data also contain a questionnaire, where we use the part of information related to the analysed stimulus1 and stimulus11. Specifically, we consider the listener’s self-rating of the concentration, prior knowledge, interest and understanding of the story, as well as the sympathy to the appointed speaker, the speaker’s narrative quality and attractiveness. The summary is shown in Table A in S1 File.

### Mapping EEG data to network and filtering relevant links

Different methods are in use for mapping the brain activity signals to a graph, see review articles [3, 42]. Each recording method and, consequently, the networks extracted from it have certain limitations. For example, fMRI has good spatial resolution, and the network nodes have anatomical locations in the brain, but the low temporal resolution to detect the neuronal activity. On the other hand, the EEG recordings are at the right temporal resolution, but the network’s nodes represent the locations of the electrodes on the scalp. However, “by invoking a general operational principle of complex networks analysis” [3], both of these microscopically distinct networks contain relevant information about the macroscopic organisation of brain function.

Here we apply the methods of correlation matrix that was widely applied for mapping EEG signals [8, 43], stock market data [44], gene expression data [45, 46] and traffic signals [47]. In this case, correlations among EEG signals describe functional connectivity patterns between different brain areas, whose activity is recorded in 63 points at the scalp. Thus, the data consists of 882 time series. The length each time series is 83499 time steps in stimulus1, and 120911, in stimulus11, where one time step corresponds to 1/500 sec. According to [41], the interesting correlations are expected when the delay 12.5s (6250 time steps) is considered between speaker’s and listener’s time. In this case, we skip the first 6250 points in the speaker’s time series and the last 6250 time points in the listener’s. Else, the speaker–speaker and listener–listener correlations are determined without any delay. First, the Pearson’s coefficient is computed for each pair (*A*_*i*_, *B*_*i*_) of time series

$$C_{AB}=\frac{1}{N-1}\sum_{i=1}^{N}\left(\frac{A_{i}-\mu_{A}}{\sigma_{A}}\right)\left(\frac{B_{i}-\mu_{B}}{\sigma_{B}}\right)\;,$$ (1)

where *μ* and *σ* are the mean and standard deviation of the corresponding time series. Further, to separate the strong positive correlations, which are relevant in this context, we use the filtering algorithm described in [45, 46, 48]; the algorithm enhances those matrix elements *C*_*ij*_ that have a similar correlation pattern with the rest of the matrix elements while diminishes those with a dissimilar patterns. First we map *C*_*ij*_ to the range [0, 1] using *CP*_*ij*_ = (*C*_*ij*_ + 1)/2. Then, each element is multiplied *CP*_*ij*_ → *F*_*ij*_ *CP*_*ij*_ by the corresponding factor *F*_*ij*_, which is computed as Pearson’s coefficient of the rearranged matrix elements from row *i* and column *j* as follows: {*C*_*ij*_, *C*_*i*1_, *C*_*i*2_, …*C*_*ij*−1_, *C*_*ij*+1_, …*C*_*iN*_} {*C*_*ji*_, *C*_*j*1_, *C*_*j*2_, …, *C*_*ji*−1_, *C*_*ji*+1_, …*C*_*jN*_}. The filtering factors *F*_*ij*_ are shown vs. the corresponding correlation coefficients in Fig A(a) in S1 File. The resulting filtered correlation matrix is also transfered to a binary adjacency matrix of the graph by retaining the correlations larger than a threshold value and inserting units for the retained edges.

### Standard graph-theory measures & network communities

In graph theory [49], a *graph* is a mathematical object consisting of nodes and edges connecting the nodes; to characterise the graph structure, some measures [42, 50] are computed, here termed the *standard graph measures* to emphasise the distinction between the algebraic-topology measures, defined below. Given the specific type of graphs, for this work we determine the diameter of the graph *d*, the graph density *ρ*, the average degree 〈*k*〉 and path length 〈ℓ〉, and the clustering coefficient Cc, as well as the community structure.

*Communities* on a network are identified as densely connected subgraphs; these are groups of nodes in which each node has more connections with the other members of the group than with the nodes outside of the group. To determine the network’s community structure [51–58] in these particular type of networks, we use the appropriate method based on the maximum modularity [59] (see also Discussion).

### Algebraic topology of graphs: quantifying higher-order topological complexes by Q-analysis

**Fig 2:**
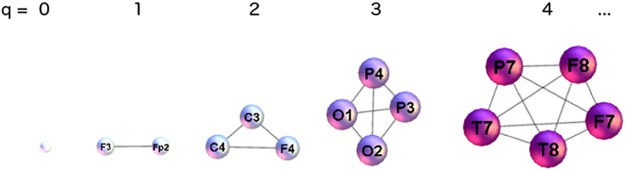
An illustration of higher-order structures (cliques) corresponding to the indicated topology level *q*.

Beyond graph theory, the algebraic topology of graphs [60–62] studies the higher-order topological spaces termed simplices and simplicial complexes to which the nodes and links appear to organise. In the clique complex methods [63, 64] that we use here, simplices are recognised as cliques of different orders *q* = 0, 1, 2⋯*q*_*max*_, see Fig 2. Here, *q*_*max*_ denotes the highest order clique that occurs in the network.

Starting from the adjacency matrix of the graph, the algorithm identifies cliques at each topology levels *q* = 0, 1, 2⋯*q*_*max*_ as well as the identity of each node that belongs to a particular clique. These data are stored as a matrix (MC matrix of cliques and the corresponding nodes). They provide information about how different cliques are interconnected, i.e., via shared nodes, links, triangles, or other cliques of a lower order (shared faces). Based on *Q*-analysis [65–67], the higher-order combinatorial structures are quantified by the structure vectors of the graph:

- First structure vector (FSV) components {*Q*_*q*_} represent the number of *q*-connected classes;
- Second structure vector (SSV) components {*n*_*q*_} identify the number of simplices of the degree *q* and larger; hence, higher values of SSV at higher levels indicate a more complex structure of the graph.
- Third structure vector (TSV) components, derived from FSV and SSV, are introduced to explicitly describe the quality of the interaction among simplices at each topology level; here we use [68] the components ${\hat{Q}}_{q}=1-Q_{q}/n_{q}$, which determine the degree of connectivity at each topology level.

The information stored in MC matrix also allows for an analysis of node’s structure vectors [68], whose components $\left\{Q_{q}^{i}\right\}$ indicate the number of simplices of the order *q* to which a particular node *i* belongs; then the node’s topological dimension $dim\left(Q_{i}\right)=\sum_{q}Q_{q}^{i}$ determines the relevance of each node in its network surrounding. Similarly, the occupation probability $p_{q}^{i}=Q_{q}^{i}/\sum Q_{q}^{i}$ of the topology level *q* and the related entropy *S*_*Q*_(*q*) measure can be determined [69].

### Comparing SB networks: Links overlap

Statistical measures of (dis)similarity between a considered pair of functional single-brain (SB) networks are quantified. In particular, we identify the fraction of the links *E* that are identical in both networks relative to the total number of connections in both networks. That is, for a pair of the listener’s single-brain-networks, the overlap measure is given by

$$O\left(L_{ix},L_{jy}\right)=\frac{E\left(L_{ix}\right)\cap E\left(L_{jy}\right)}{E\left(L_{ix}\right)\cup E\left(L_{jy}\right)}$$ (2)

where *i*, *j* ∈ {1, 2} denotes the group to which the listener belongs, *x*, *y* ∈ 1, …, 6 and denotes the listener’s identity number. Here *L* stands for listener; the analogous expression applies to any pair of *L*−−*S* and *S*−−*S* networks.

### Comparing SB networks: Graph-edit distance (GED)

GED counts the number of links that have to be deleted so that the two matrices become equal [70]. These counts are then used to position all listeners relatively to the two speakers in a 2D-coordinate space. We set *S*_1_ in the origin (0, 0), while *S*_2_ on the coordinate (*s*_12_, 0), where *s*_12_ is the GED between *S*_1_ and *S*_2_. The (*x*, *y*) coordinates of a listener are then calculated using trigonometric functions, where the length of the sides of the triangle are computed GED between the listener and both speakers.

### Randomisation of SB networks

We apply two procedures to randomise the network connections. In Random-K, the procedure preserves the node’s degree. Starting from the original list of links, considered as oriented, for each link we find a randomly selected link in the list of all links and cross switch the out-linking between the corresponding pairs of nodes. We repeat the process until each link is switched at least once. We also apply the fully randomised procedure, here termed Random-L, which preserves the total number of connections in the network. In this case, while cutting a link from the original list, we insert a new link among a randomly selected disconnected pair of nodes.

## Results

### Correlation matrix and networks of speakers’ and listeners’ EEG signals

**Fig 3:**
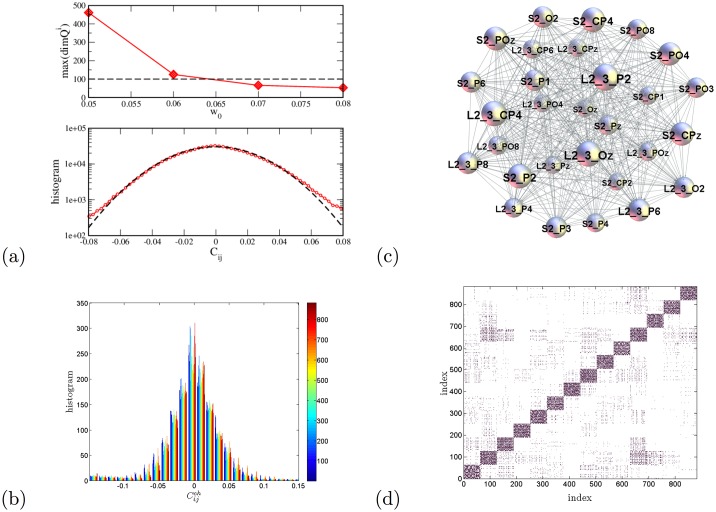
Mapping multi-brain correlations. (a) Maximum topological dimension plotted against various thresholds *w*_0_ (top panel) stabilises near the selected threshold 0.06. The middle part of the histogram of correlation coefficients averaged over 882 channels (lower panel); the fit by the normal distribution, dotted line, deviates from the data for the correlations larger than 0.06. (See the histogram for the whole range in Fig A(b) in S1 File). (b) The central part of the histograms for different channels. For a particular channel, the correlations with other 881 channels are marked by the corresponding colour indicated in the colour map; the presence of colours over different bins suggests that all channels obey a similar distribution. (c) An example of higher-order structure involving the signal locations at two scalps—the speaker’s S2 and the listener’s *L*_2−3_. (d) The adjacency matrix of the multi-brain network of the two speakers and 12 listeners with the threshold *w*_0_ = 0.06. The order of indexes is as explained in the text.

We analyse two different stimuli from the corpus of the previously recorded and pre-processed data of Ref. [41]. A full description of the analysed datasets is given in Methods. In each stimulus, we consider EEG signals recorded during the session at 63 locations on the scalp of two speaker and 12 listeners, in total 882 signals. The correlations among each pair of signals are computed as Pearson’s coefficient. Between the speaker’s and listener’s events, we take into account the characteristic delay of 12.5s, which was observed and related to the processing of semantic content in the original work [41]. The equal-time correlations are considered among signals of the pairs of listeners. The correlation matrix is filtered (see Methods) to diminish the potentially redundant correlations. Finally, a threshold value (in this work *w*_0_ = 0.06) is applied to remove weak correlations that are assumed to be normally distributed, Fig 3a and 3b.

**Fig 4:**
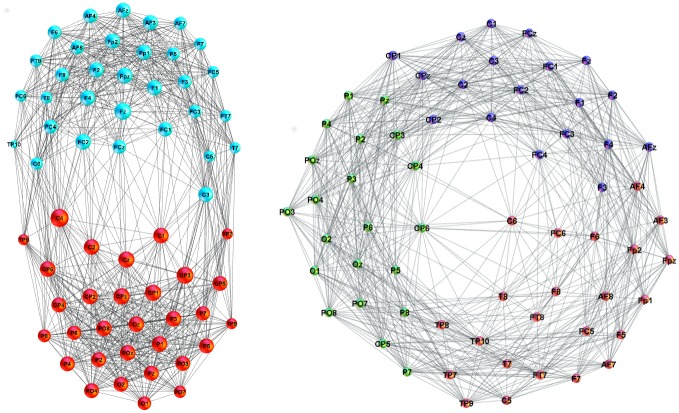
Functional brain network from EEG signals. (left) An example of network mapping the functional brain connections recorded by EEG signals on the speaker’s scalp (labels). The applied threshold *w*_0_ = 0.06. The colour of nodes indicates two identified communities—frontal (F) and parietal (P). (right) Loss of the F/P community structure at a lower threshold *w*_0_ = 0.05 in the correlation matrix.

To select the right threshold, we observe the criteria that the modular decomposition, as chief feature of functional brain networks [51, 52], shows consistency in the multi-brain networks for the values above the selected threshold. Also starting from this threshold value, each single-brain connectivity splits into a frontal and parietal cluster, the structure characteristic to awaken state according to Ref [14], whereas they may join to a single cluster when the threshold is lowered, see Fig 4. Moreover, we checked that the cross–brain connections around this threshold form a sparse network but sufficiently connected to ensure nontrivial structures, as the example in Fig 3c; the occurrence of large simplicial complexes tested by the node’s topological dimension stabilises around the selected threshold value (see Fig 3a,top). The adjacency matrix of a binary multi-brain graph is then constructed, i.e., *A*_*ij*_ = 1 when the matrix element *w*_*ij*_ > *w*_0_ and zero otherwise, and shown in Fig 3d. In this aggregate multi-brain network, *the single list of indexes indicates the standard abbreviations of the 63 EEG scalp locations belonging to, respectively, speaker S1, speaker S2, then two groups of listeners* *L*_1−*k*_ *and* *L*_2−*k*_, *k* = 1, 2, ⋯6. Thus, the diagonal blocks in the adjacency matrix represent the 63 × 63 connectivity matrices related to each brain in the above-defined order. Whereas, each off-diagonal block contains the corresponding brain-to-brain correlations.

Here, we first analyse the structure of each diagonal block and some selected cross-correlations in the aggregate network in Fig 3d. Then we turn to the analysis of the whole multi-brain network, which allows for quantifying the speaker’s impact onto the listener’s brain activity patterns. Each diagonal block of the aggregate adjacency matrix in Fig 3d represents a functional brain network related to a particular participant, as described below. In the present context, these separate subgraphs are termed single-brain-networks (SBN) to distinguish them from the studied multi-brain structures. Fig 4 shows examples of SBN obtained at two different thresholds.

### Single-brain networks (SBN) of speakers and listeners: Quantifying the topological differences

**Fig 5:**
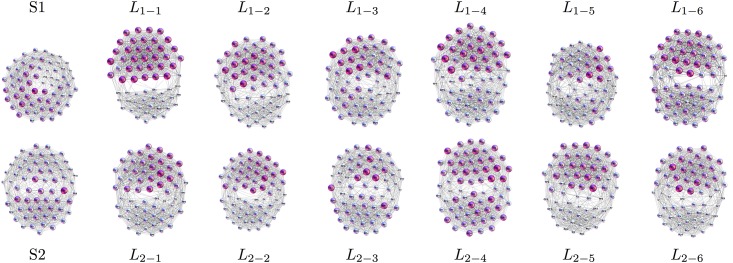
SBN for speakers and listeners in stimulus1. In each network, nodes represent different scalp locations (labels) while each link indicates the positive correlation that exceeds the threshold among the corresponding pair of EEG signals. Remarkably, each connectivity network visually splits into two clusters, which can be identified by the location labels as frontal (upper) and parietal (lower), also confirmed by the community detection analysis. See an example of a larger picture in Fig 4.

In Fig 5 the single-brain networks representing the correlation of the EEG signals on the scalp of both speakers and the listeners in both groups are shown. As we stated in the Introduction, the focus is on the occurrence of higher-order structures in SBN and other relevant subgraphs of the multi-brain network. In this respect, the topology levels and the corresponding vectors defined in Methods are first computed for all SBN, as the relevant units of the multi-brain graph. Before turning to the topology analysis, for comparison, we also determine the standard graph-theoretic measures for all SBN; they are summarised in Table 1. Further, we analyse the (dis)similarity between these functional connections in all pairs of SBN.

**Table 1:** Graph-theoretic characteristics of single-brain connectivity networks. Standard graph measures denoted in the first column (see Methods) for each SBN of the two speakers and two groups of listeners listed in the top row; data are for the case of stimulus1. For comparison, we also show the number of topology levels *q*_*max*_ and the number of cliques of the highest order in the corresponding network.

SBN	S1	S2	*L*_1−1_	*L*_1−2_	*L*_1−3_	*L*_1−4_	*L*_1−5_	*L*_1−6_	*L*_2−1_	*L*_2−2_	*L*_2−3_	*L*_2−4_	*L*_2−5_	*L*_2−6_
<*k*>	25.8	27.7	29.0	28.1	27.2	27.3	26.9	27.7	27.4	27.2	26.1	27.6	28.0	27.7
<ℓ>	1.64	1.63	1.71	1.62	1.63	1.66	1.65	1.70	1.66	1.65	1.69	1.65	1.63	1.67
*Cc*	0.676	0.743	0.847	0.745	0.717	0.749	0.726	0.785	0.759	0.757	0.736	0.759	0.752	0.763
*ρ*	0.416	0.448	0.468	0.453	0.440	0.441	0.435	0.447	0.442	0.441	0.421	0.444	0.452	0.447
*mod*.	0.307	0.368	0.412	0.346	0.346	0.370	0.355	0.414	0.382	0.382	0.379	0.390	0.352	0.373
*n*.*c*.	3	2	2	2	3	2	3	2	2	2	2	2	3	3
*q*_*max*_	16	19	29	23	19	23	20	22	18	22	20	20	22	24
*n*_*q*_*max*__	1	6	1	1	2	1	4	1	1	1	2	5	6	1

**Fig 6:**
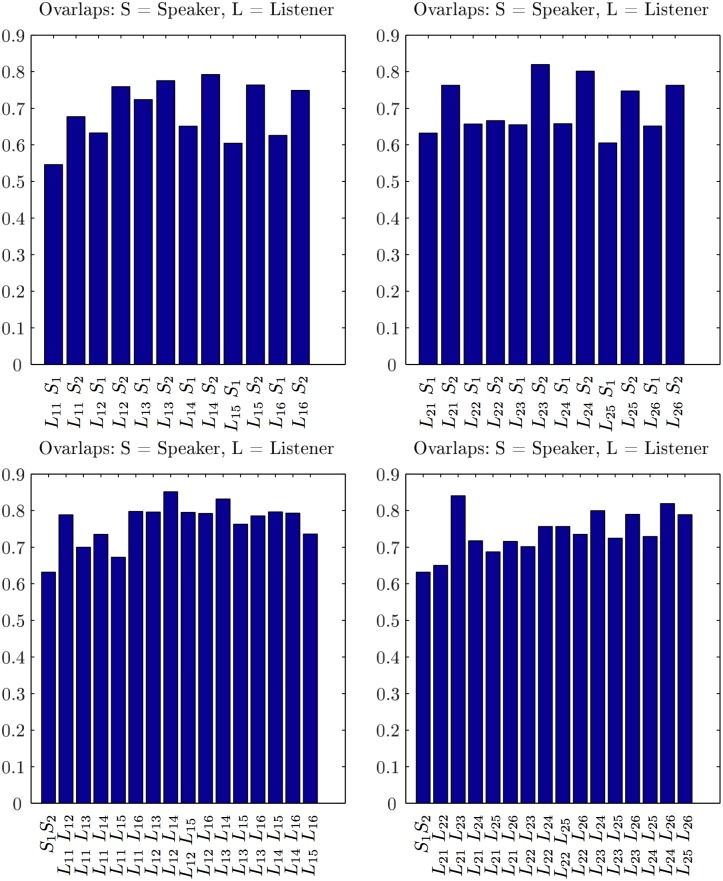
The similarity of single-brain networks in stimulus1. SBN overlaps between each listener in group 1 with speakers S1 and S2, the left panel, and each listener in group 2 with S1 and S2, right panel. Bottom left and right panels show the SBN overlaps between pairs of listeners in the group 1 and group 2, respectively. For a comparison, the SBN overlap between two speakers is also shown (the first bar in each bottom panel). Note that all overlaps are significantly larger than in the corresponding randomised models (see Figs C and D in S1 File).

**Fig 7:**
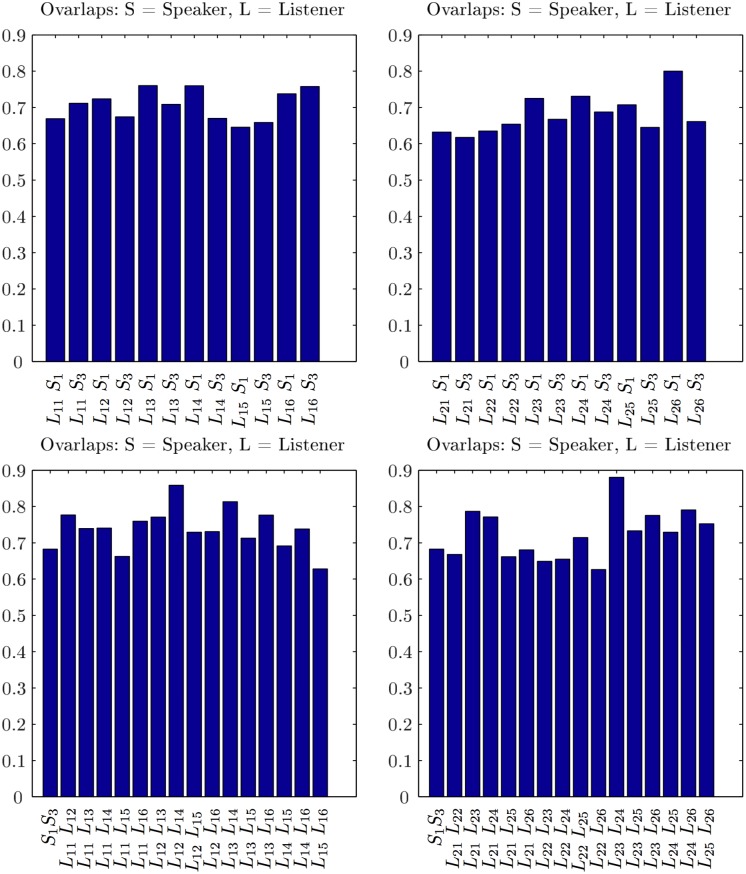
The similarity of single-brain networks in stimulus11. Link overlaps of the two listener’s groups, explanations as for Fig 6, but with the speakers S1 and S3, respectively.

Comparing two networks is widely used in the literature to uncover the nodes and links that are responsible for a particular pattern, e.g., disease-related connections [53], or to infer the relevance of a particular link or a node [54]. In the present context, we expect that speakers and listeners use different activation of the brain areas to process and perceive the semantic contents during the communication. Thus, the corresponding brain activity patterns, reflected in several positively correlated EEG channels, can be differentiated through the differences in the SBN’s topology. First, we compare the pairs of SBN by examining the presence/absence of a particular link (the correlated pair of channels). In this regard, the differences in the activity patterns are observed among both speakers and, evoked by the stimulus, among the listeners within the same group as well as across the groups. Here, we quantify these differences using two methods of graph comparisons. First, we determine the extent to which the edges overlap (see Methods) in each pair of single-brain networks. The resulting statistics is displayed in Figs 6 and 7 for the situation in stimulus1 and simulus11, respectively. Furthermore, the occurrence of the excess links leading to a finite distance between the pairs of single-brain graphs in the topological space is expressed by graph-edit-distance, as described in Methods.

#### Link overlap statistics

The link-overlap statistics expresses the degree of similarity between each compared pair of SBNs; it is computed according to the formula (2) in Methods. In Fig 6, we show the outcomes for the overlaps for the listeners of both groups in stimulus1 with both speakers, as well as the overlaps among the pairs of listeners in both groups, exposed to the same input. Similarly, the overlaps for stimulus11 are shown in Fig 7.

As the Fig 6 shows, the brain activity patterns of both groups of the listeners in stimulus1 express a larger similarity to speaker S2 than to speaker S1. It is also interesting to notice the overlaps among pairs of listeners in each group. Noticeably, the overlap between SBN of the two speakers is low (minimal overlap in the entire set). The situation is just opposite in stimulus11, where the same speaker S1 features, however, narrating a different subject. While the overlap between the two speakers is larger than in stimulus1, four listeners in group 2 have a better overlap with speaker S1 than with the attended speaker S3. This situation manifests in a specific heterogeneity of the overlaps between the listeners in group 2. It is important to stress that, while the edges overlap varies among different pairs, it always stays significantly above the corresponding values for the randomised models. The overlaps obtained for the pairs of randomised SBN are calculated and given in Fig C in S1 File, for the degree-preserving randomization, and Fig D in S1 File, for the fully randomised graphs with the same number of links.

#### SBN’s distance between listeners and speakers

**Fig 8:**
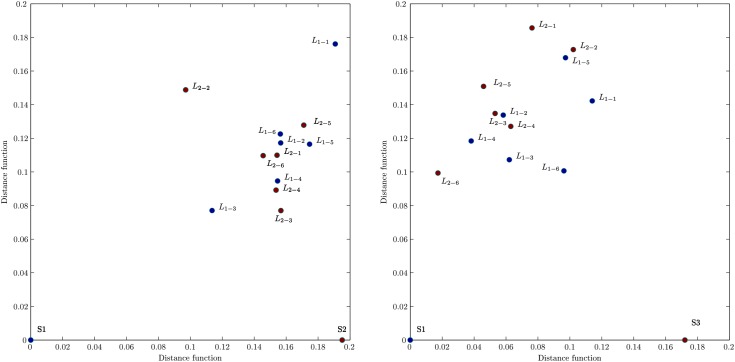
Graph edit distances between listeners and speakers. In stimulus1 (left) and stimulus11 (right), the speaker S1 is placed in the origin and the speaker S2 (S3) at the corresponding distance along the x-axis while the coordinates of the listeners of both groups are shown in the distance plane. Concerning GED, both groups of listeners systematically appear closer to a “right” speaker (S2 in stimulus1, S1 in stimulus11) according to the listeners’ subjective ratings.

The excess links, which are present in an SBN but do not overlap with another SBN, represent a measure of distance between these networks in the topology space. Applying the graph edit distance (GED), as described in Methods, we first compute the distance between SBNs of speaker S1 and speaker S2. Then the distances of each listener from both speakers are calculated and presented by a point in the distance plane in Fig 8; two panels are for the stimulus1 and stimulus11, respectively. For a better comparison, both groups of listeners are plotted on the same graph.

The following features of the distance graphs are interesting. First, in the stimulus1, the majority of the listeners from both groups are closer, suggesting a larger similarity in the brain connectivity patterns, to the speaker S2, than to the speaker S1. Excluding the listeners *L*_1−1_, *L*_2−2_ and, to some extent, the listener *L*_1−3_, the listeners of both groups form a cluster in the distance plane. Notice that, by definition of GED, the closeness of two listeners in this graph is referring to the similar *fraction* of the removed links. However, by comparing the exact links, the two listeners may have a finite distance from each other.

These properties of the distance plots are in good agreement with the histograms of the pairwise overlap in Fig 6. Namely, for the stimulus1, except for *L*_2−2_ and *L*_1−3_, the speaker’s S1 overlaps with both groups are worse than the overlaps of the speaker S2 with the listeners in both groups. Moreover, the listeners in the centre of the cluster, for instance, *L*_1−2_ in group 1 and *L*_2−6_ in group 2, have similar overlaps with the remaining members of the group. Further comparisons of the listeners, for instance, *L*_2−3_ and *L*_1−4_, who are quite close in the distance space but have different appointed speakers, reveals different cross-brain correlations, see later.

In the stimulus11, the same speaker S1, here narrating a different type of story, attracts more attention of the listeners in both groups than the new speaker S3. In this context, some striking examples are the listener *L*_2−6_ and *L*_1−6_, cf Fig 8. Note that in this case the listeners *L*_1−2_, *L*_1−3_, *L*_1−4_, *L*_2−3_ and *L*_2−5_ also form a group in the distance plane. In the analogy with the above-discussed stimulus1, these findings of the distance graph are in agreement with the corresponding overlaps in Fig 7 for stimulus11. Hence, the impact of a speaker may strongly depend on the narrating subject; formally, its corresponding brain activity results in a different network (see more details in Discussion). These features of SBNs are also reflected in the appearance of higher organised structures, as discussed in the following sections.

It is interesting to compare these objective graph-theoretic measures with the subjective experience of each listener; the self-reported ratings of self-concentration, quality of the speaker and interest in the story related to the stimulus1 and stimulus11 are summarised in Table A in S1 File. While in both stimuli, all listeners reported no prior knowledge of the story, their interest varies in correlation with the self-reported narrative quality of the speaker. The listener’s report of weak sympathy to the speaker, low speaker’s narrative quality and attractiveness (i.e., speaker S1 in stimulus1, speaker S3 in stimulus11, cf. Table A in S1 File) correlates well with the increased distance between speaker–listener SBN and low overlap. Oppositely, self-reported sympathy to the speaker, qualifications as attractive and good narrator (i.e., speaker S2 in stimulus1 and speaker S1 in stimulus11, cf. Table A in S1 File) agree well with the increased SBN overlaps and reduced topological distance, Figs 6, 7 and 8.

#### Topological spaces of single-brain networks and inter-brain linking

**Fig 9:**
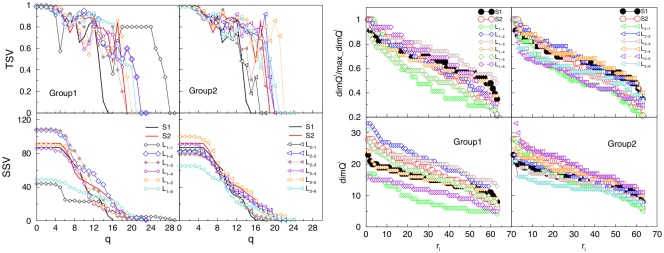
Topology measures of the SBNs of speakers and two groups of listeners for stimulus1. Components of the second (SSV) and third (TSV) structure vectors (left panels), and the ranking distributions of the nodes’ topological dimensions (right panels). The full lines indicate the corresponding measures of the speakers.

As mentioned in the Introduction, we anticipate that the key features of the brain activation of an individual during social communication are contained in the hierarchical organisation of links between involved brain areas, which leads to the occurrence of topological complexes. In the multi-brain graphs, the occurrence of higher-order structures is manifested in two ways: (i) the appearance of hierarchical organisation along different topology levels in each SBN, and (ii) the inter-brain correlations leading to a nontrivial community structure of multi-brain graph as well as the hierarchical organisation of these communities. In comparison with known heuristic approaches for hierarchical communities [52, 55], here we rely on mathematically strict definition of simplexes and simplicial complexes, as described in Methods. First, we compute the structure vectors defined in Methods to describe the higher-order structures in the individual brain connections both for speakers and listeners. In Fig 9, we show the results of the first and third structure vectors for each of 14 single-brain networks in the case of the stimulus1.

Performing the Q-analysis of each SBN, the components of the structure vectors *Q*_*q*_ and *n*_*q*_ are computed at each topology level *q* = 0, 1, ⋯*q*_*max*_, where *q*_*max*_ is the order of the highest clique found in the corresponding brain connectivity graph. Combined with *Q*_*q*_, the components of the third structure vector are deduced, which quantify the connectivity among different cliques at each topology level. In this respect, both speakers, as well as the two groups of listeners, differ, as displayed in Fig 9. As a rule, the highest topological level in the case of listener’s SBN connectivity exceeds one of the speaker’s. Moreover, SBN of the speaker S2 exhibits higher organisational complexity at all levels *q* > 10 than the speaker S1. The results for the majority of the listeners in the group *L*_2−*k*_ are quite coherent and mainly follow the structures found in SBN of the speaker S2. However, in the group *L*_1−*k*_ the listeners’ structures exhibit larger deviations from the speakers’ either at small or at large *q*. The striking example is the listener *L*_1−1_, whose pattern of connections exhibit a much lower number of small complexes but also a certain number of vast complexes reaching at *q*_*max*_ = 29. This situation implies that, in contrast to all other participants in the stimulus1, in the case of the listener *L*_1−1_ a sizeable number of unusual connections are present, which enable the formation of cliques of the order from 24, ⋯30, cf. SBNs in Fig 5. Note also that in the graph-distance measure, the listener *L*_1−1_ is far away from the both groups.

The analysis is complemented by the ranking distribution of 63 nodes in each SBN, according to the node’s topological dimension, right panels in Fig 9. Again, the lines related to different listener’s SBN suggest a higher heterogeneity of these networks for the listeners in group 1 than the group 2. These quantitative topology measures correlate well with the listeners’ qualitative experience, cf. Table A in S1 File, showing a wide variation of the concentration, interest, and understanding as well as a weak sympathy to the speaker and low rates of his attractiveness and goodness.

**Fig 10:**
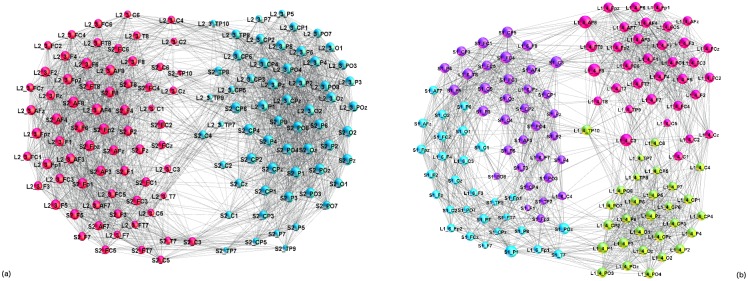
Structure of inter-brain linking. Superbrain network of the speaker S2 and the listener *L*_2−3_, illustrating proper coordination, (left), and a weakly connected two-brain structure of the listener *L*_1−4_ and the speaker S1, corresponding to wrong coordination (right). Different colour of nodes indicates the identified functional communities. The node’s labels belong to the unique list of 882 scalp locations of all participants; for example, *L*2_−_3_−_*TP*9 and *S*2_−_*F*7 indicate the channel “TP9” on the scalp of the listener 3 in group 2, and channel “F7” of the scalp of the speaker S2, respectively. See also the overlaps in Fig 6 and distances in Fig 8 for these pairs.

Apart from the diagonal blocks of the adjacency matrix in Fig 3, the off-diagonal matrices exhibiting the inter-brain connections provide valuable information about the communication impact (speaker–listeners) as well as the brain-function synchronisation under the same input stimulus (listener–listener correlations). These connections contribute to a nontrivial structure of the multi-brain network. In Fig 10, we show two representative examples visualising differences in two-brain networks of a listener and a speaker. In one case, *L*_2−3_ well correlates with the speaker S2. The corresponding two-brain network has 630 cross-links and an original structure with two communities, each of which contains the scalp locations of both individuals. This super-brain structure confirms a real focus of the listener *L*_2−3_ to the speaker’s S2 story, in full agreement with the corresponding distance and SBN overlap measures for *L*_2−3_ and S2 discussed above, as well as the listener’s self-reported experience in Table A in S1 File. Oppositely, the two-brain network of the listener *L*_1−4_ and the speaker S1 exhibits very few cross-links (57) and a community structure featuring separate brains. These results also agree well with the self-reported low concentration, uninteresting and confusing story, and bad qualities of the speaker (see Table A in S1 File).

### Communities and topological spaces in multi-brain network

The interbrain synchronisation, which is often observed during social communications [31, 32, 38, 39], is also expected in the analysed spoken communication experiment; in the present context, it is embedded in the structure of the entire multi-brain network. Here, we perform a formal analysis of the multi-brain graph to describe the social impact on the brain activity of each participant. Moreover, we analyse the appearance of mesoscopic structures (communities) that involve scalp locations over several brains, as well as the hierarchical organisation of a particular community graph.

**Fig 11:**
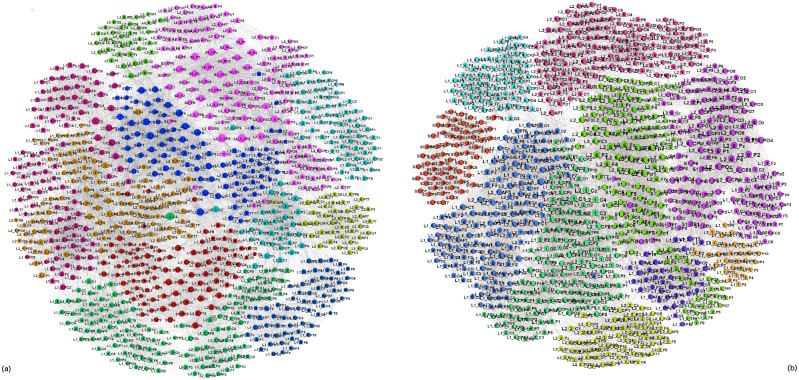
Multi-brain networks. Communities, marked by different color, of nodes in the whole multi-brain network in stimulus1 (a), and stimulus11 (b). The nodes’ labels comprise the unique list of 882 scalp locations of all participants, as explained in the caption to Fig 10.

**Fig 12:**
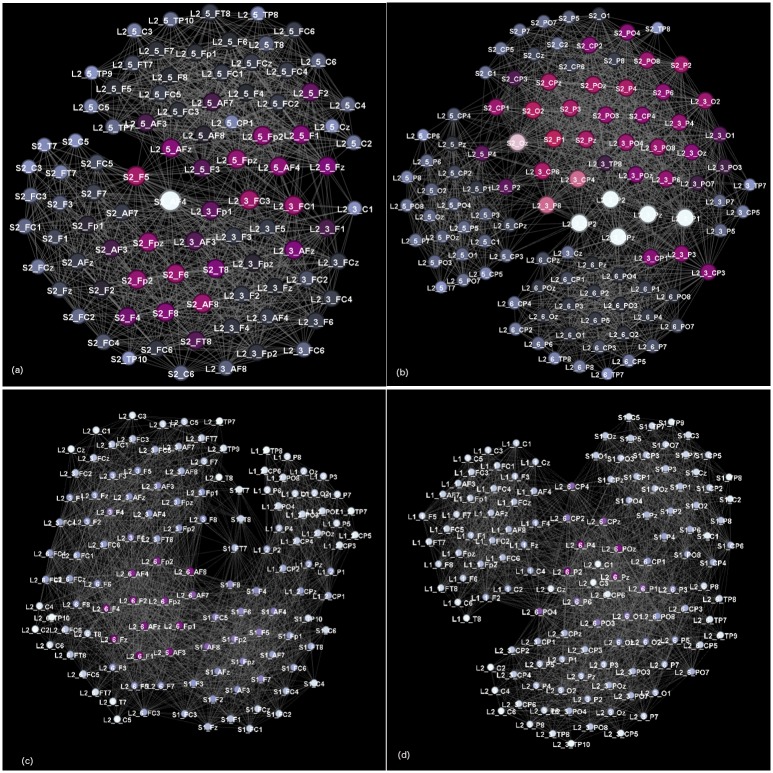
Speaker-related communities occurring in multi-brain network. Two communities dominated by frontal and parietal lobe locations are shown as separate graphs in stimulus1, (a) and (b), and in stimulus11 (c), and (d).

In general, the presence of communities is relevant for the synchronisation of stochastic processes taking part on the graph [52, 56]; the characteristic time scale of the coherence dynamics on different communities is directly related with the lowest eigenvalues of the Laplacian operator related with the graph’s adjacency matrix while the corresponding eigenvectors localise on these communities [57]. Here, the activity patterns, involving different areas in the multi-brain graph, lead to the enhanced correlations and dense subgraphs or communities that involve scalp locations of several listeners and a speaker. The community structure of the multi-brain network both for simulus1 and stimulus11 are shown in Fig 11. In each case, there are several communities of different sizes. While some single-brain network (as the listener *L*_1−1_ and *L*_2−2_ in the case of stimulus1, and similarly, speaker S3 in stimulus11) comprises a separate community, the majority of the identified communities are cross-brain type involving parts of the nodes in SBN of different participants. Two such communities, related to the speaker S2 in simulus1 are shown separately in Fig 12a and 12b. Similarly, examples of the communities related to the speaker S1 in stimulus11 are shown in Fig 12c and 12d, respectively. It is interesting to stress that typically frontal scalp areas across different brains often form a separate community while parietal areas belong to another (here termed F- and P-community), cf. labels in Fig 12a–12d. A similar structure of the communities occurs in the two-brain network in Fig 10a in a direct relation to a right speaker–listener coordination.

#### Synergy in the multi-brain communities

**Fig 13:**
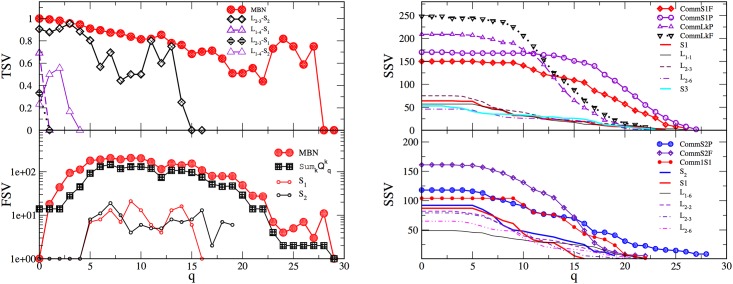
Topology vectors of multi-brain graphs. Left panels: Components of the first (FSV) and the third (TSV) structure vectors plotted against the topology level *q* for the whole multi-brain network and for some its subgraphs, as indicated in the corresponding legends. The additive components of the FSV allow a comparison of the whole MBN with the sum of the corresponding component of each participating SBN, the line is indicated by $Sum_{k}Q_{q}^{k}$, where *k* runs over all listeners and the two speakers in stimulus1. TSV of cross-graphs in two-brain networks from Fig 10 and their counterparts are shown. Right panels: Components of the second (SSV) structure vector of the largest four communities in stimulus11 (top) and three communities in stimulus1 (bottom). For comparison, the values obtained for the corresponding SBN of the speakers and listeners participating in these communities are also shown.

The results of algebraic topology analysis of the entire multi-brain network (MBN) and its largest communities are given in Fig 13. First, we compare the (additive) components of the first structure vectors FSV of the whole MBN with the sum of the components of all SBN. Remarkably, the MBN exhibits a more complex structure, i.e., higher values at all topology levels, which can be attributed to the contributions of inter-brain subgraphs, cf. the adjacency matrix in Fig 3. Hence, this feature of the MBN is a good quantifier of the social impact among the communicating brains. Similarly, the third structure TSV shows that the simplexes at all topology levels up to *q* = 28 are strongly interconnected in the MBN. In this context, the TSV of the corresponding cross-brain subgraphs suitably quantifies the speaker–listener coordination. The results of TSV for the cross-links in the two-brain network in Fig 10 show that the proper coordination among the listener *L*_2−3_ and the speaker S2 corresponds to a topologically rich structure; in contrast, a weaker or improper coordination between *L*_1−4_ and the speaker S1 results in a much simple topology.

The two frontal- and parietal-communities from Fig 12 are associated with the speaker S2 and involves several listeners. The topology analysis of these and some other communities (the SSV is shown in Fig 13) reveals an interesting structure. In general, in the situation of proper focus with a speaker (S2 in simulus1, S1 in stimulus11), a clear differentiation of F-based and P-based communities is found. Among these, P-based communities exhibit a richer structure, especially in the presence of higher order simplexes. In contrast, the situation of weak focus with the appointed speaker (S1 in simulus1, S3 in stimulus11), the speaker-related community involves a mixture of different SBN nodes of the speaker and the dedicated listeners. The SSV of such communities resembles an SBN the speaker’s SSV. Also, the absence of a proper coordination with the speaker, several listeners appear to form a community, where also F- and P-based structures are present, but they are comparable in the topological complexity and much simpler than the speaker-based communities, cf. Fig 13.

## Discussion

By mapping a hyper-scanning dataset onto multi-brain network, we developed a systematic approach to quantify the differences in the brain activity patterns and inter-brain coordination during social communications. Our analysis of the representative spoken communications datasets (two stimuli, each consisting of the simultaneous EEG recordings of 12 listeners and two speakers narrating different stories) confirms the leading idea of this work. Namely, the brain activation of each participant (depending on its role, communicated contents, and other factors) manifests in particular interconnections of the affected brain areas; these interconnection patterns lead to significant higher-order structures in the related brain network and inter-brain graphs, which are adequately described by the algebraic topology measures. Precisely, the hierarchical structure of the scalp connectivity network and inter-brain graphs are quantified by the number of topology levels *q*_*max*_ given by order of the largest clique found in the network, and the ways that the cliques organise into larger complexes by sharing nodes at lower levels from *q* = *q*_*max*_ − 1, ⋯1.

Assuming that the fluctuations of EEG signals on the scalp suitably reflect the underlying brain activity, the approach allows analysing the fine-grain correlations (63 channels) of each participant, as well as cross-correlations between different brains, and the aggregate multi-brain graph of two speakers and twelve listeners. The major advantage of this approach is that (even without knowing exact relationships between the correlations of the measured signals and potentially affected deeper brain areas), a comparative analysis of various networks provides a good measure of the *differences in the underlying brain activity*. This type of analysis combines well with the statistical features as well as with the listeners’ self-rating experience during the communication.

**Fig 14:**
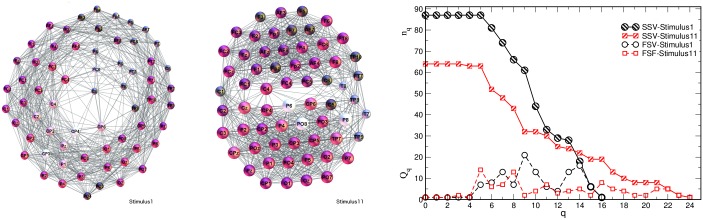
Subject-specific brain activity patterns of the speaker S1. From left to right, SBNs represent EEG correlation patterns of the speaker S1 narrating a fairy tale (in stimulus1) and giving instructions (in stimulus11), and the components of the first and the second topology vector of these SBNs.

**Fig 15:**
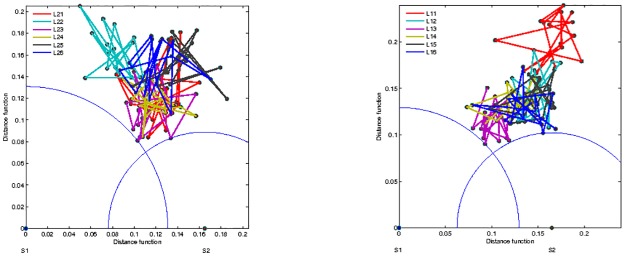
Evolution of the brain-to-brain distance. The timeline (16 frames) of the GED between brain activity networks of the listeners in group 1 (left) and in group 2 (right) from both speakers S1 and S2 is shown for the stimulus1. Each circle indicates the minimum distance from the corresponding speaker that occurred during the process.

According to these higher-order topology measures, we discovered some new features of the brain activity networks, which are not accessible to the conventional statistical methods and standard graph theory. In particular:

- *Significant differences between SBNs* occur across the listeners and speakers, and are quantified by the topology vectors. Across the listeners’ groups, the degree of heterogeneity strongly correlates with the increased distance to the appointed speaker. We also find $q_{max}^{L}>q_{max}^{S}$ in all studied examples, suggesting that the listener’s brain activity results in a more complex architecture than the speaker’s. In agreement with the statistical analysis in [41], this fact relates to the processing of semantic content in the presence of noise. A more detailed analysis reveals the excess links in the listener’s SBN; these links correspond to the coherence between a set of different EEG channels, not occurring in the speaker’s network. Moreover, these topology quantifiers accurately distinguish the patterns of the brain activity of the same speaker while narrating different subjects. Fig 14 displays the differences between corresponding EEG correlation networks as graphs and at each topology level for the speaker S1. Notably in contrast to the stimulus1, the number of big organised structures (for 16 < *q* < 24) occur in the case of the speaker’s narration in stimulus11, which also obtained higher ratings by the listeners.
- *The proper speaker–listener coordination* is suitably quantified by the topological similarity of their SBNs and a rich structure of the corresponding two-brain network. At lower topology levels $1<q<q_{max}^{S}$, the majority of listeners in both groups exhibit the brain activity patterns that are more similar, i.e., have better coordination, with one speaker than with the other. These topology findings are also supported by the statistics of the link overlaps and brain-to-brain distance measures, which consider *q* = 1 level, i.e., the presence and absence of each particular link in the compared networks. Interestingly enough, these findings compare well with the listener’s self-rating of the sympathy to the speaker, the speaker’s narrative quality and attractiveness, as well as the clarity and the interest of the story, as displayed in Table A in S1 File. It turns that a complex two-brain network suitably represents the case of a proper coordination. The strong frontal–frontal and parietal–parietal connections between the brains appear as two communities in a super-brain structure, cf. Fig 10a. These features are absent in the case of weak coordination, as the example in Fig 10b. Further analysis of the inter-brain correlations concerning the issues of the experimental design, questionnaire, and the semantic contents is left for the future work.
  The coordination with a right speaker evolves over time, as measured by the distance between speaker–listener SBN constructed in a sequence of time intervals, cf. Fig 15. However, there is always a gap (minimal distance) between a speaker and anyone of the listeners, in agreement to the occurrence of higher structures and extra links in the listener’s activity networks, mentioned above. It is also interesting to note that the listener’s networks exhibit a high degree of similarity, perhaps suggesting similar initial brain activity patterns, before focusing to a particular speaker.
- *F/P communities in the multi-brain networks* reveal super-brain features. At the aggregate graph level, the communities occur primarily related to a “good” narrator. These communities, as a rule, coordinate frontal brain areas of the speaker with the listener’s frontal areas, and, similarly, parietal-to-parietal. These communities regarded as separate graphs exhibit a rich structure with a large number of high-order cliques and their complexes. It is interesting to note that in each identified community structure, the parietal-based community appears to be more complex than the frontal-based one. The occurrence of speaker-related communities suggests that a significant number of channels, which are coordinated in the speaker’s brain also appear to be coordinated in the listener’s brains. In contrast, when the appointed speaker is not followed (the case of speaker S1 in stimulus1 and speaker S3 in stimulus11), some listeners appear to form communities, again connecting through frontal (parietal) channels. However, the corresponding topology of the listener’s communities is quite simpler than the topology of the speaker-related communities. In this case, the listeners exposed to the same external input synchronise their activity patterns, without having any direct communication. The observed community structure is consistent when the stimuli and the threshold values are varied. Note that the presence of community structure was confirmed as an essential feature of this type of brain networks in [52] using the local algorithm.

Quantitatively, the aggregate graph structure shows new topology features as compared to the sum of the corresponding individual measures, i.e., in the case of the additive FSV. In this way, the new structure emanating from the cross-brain connections appropriately describes the social impact onto the individual brain activity that can be captured by EEG signals.

## Conclusions

We have considered the social brain structure in two concrete examples of simultaneous EEG recordings during spoken communications (of 12 listeners and two speakers narrating different subjects) by mapping the data onto the multi-brain network and applying the methods of algebraic topology of graphs. We have shown that the topology of higher-order complexes precisely quantifies the differences in the brain activation pattern between the participants during the social communication. Furthermore, the topology provides the accurate measure for the speaker–listener coordination and the speaker’s impact onto a group of listeners. Our results also suggest that the mechanisms for super-brain functioning during spoken communications certainly involve strong frontal-to-frontal and parietal-to-parietal synchronisation in dyads. In a more general context, the study of higher-order combinatorial structures by algebraic topology techniques provides a sensitive methodology to quantify the *shifts in the functional brain networks*, e.g., under changed activity or condition. By complementing the standard graph theory methods, the algebraic topology can contribute to a more in-depth analysis of other brain imaging data.

## Supporting Information

S1 File Contains Table A, Fig A, Fig B, Fig C, Fig D. (PDF)
