# Supplementary material for: Algebraic Topology of Multi-Brain Connectivity Networks Reveals Dissimilarity in Functional Patterns during Spoken Communications
Source: PLoS One. 2016 Nov 23;11(11):e0166787. doi: 10.1371/journal.pone.0166787 (PMC5120797; doi:10.1371/journal.pone.0166787)
Supplement: S1 File — (PDF) [file pone.0166787.s001.pdf]

S1 File. Contains Table A, Fig A, Fig B, Fig C, Fig D

	concentr.	interest	understand	(un)known	sympathy	attractiveness	goodness
L11	3	2	2	6	3	5	5
L12	5	2	3	7	5	6	5
L13	3	4	5	6	5	6	6
L14	6	5	6	7	5	7	5
L15	3	6	2	7	5	6	6
L16	3	3	3	2	3	4	4
L21	2	4	2	7	2	3	1
L22	2	3	2	7	2	2	2
L23	2	1	1	7	1	2	1
L24	3	3	3	7	3	2	2
L25	1	4	1	7	3	4	3
L26	3	1	1	7	1	2	1
						Stimulus1	
L11	1	2	1	7	1	2	1
L12	3	3	4	7	4	4	4
L13	2	1	1	7	1	2	2
L14	2	1	2	2	2	5	2
L15	1	2	1	7	2	3	2
L16	2	1	1	7	2	4	1
L21	6	7	5	6	3	4	5
L22	5	5	4	7	4	4	5
L23	6	6	5	7	4	4	6
L24	6	5	4	7	3	2	5
L25	2	2	3	7	2	2	4
L26	3	5	4	5	4	4	5
						Stimulus11	

Table A: Self-rated experience of the listeners. Self-rated experience of the listeners groups L_{1k} and L_{2k}, $k = 1, 2, \cdots 6$ during the session in the stimulus1 and stimulus11, from the Questionnaire data of Ref. [41]. Left to right: self-concentration, interest, understanding and prior knowledge of the story, sympathy to the speaker, the speaker's attractiveness and narration quality (1–high, 7–low).

[41] Kuhlen AK, Allefeld C, Haynes JD. Content-specific coordination of listeners' to speakers' EEG during communication. Frontiers in Human Neuroscience. 2012;6(266):1–15. Available from: http://www.frontiersin.org/human_neuroscience/10.3389/fnhum.2012.00266/abstract

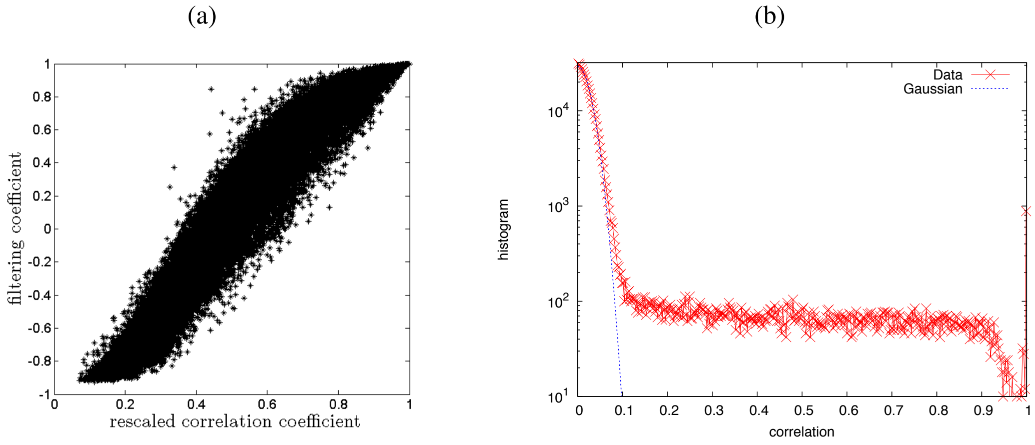

Fig A: The correlation coefficients and the filtering factors. (a) The filtering factors plotted against the shifted correlation coefficients. Note that the original correlations C_{ij} that are close to zero (close to 0.5 in the shifted scale) are filtered out, i.e., their filtering coefficients F_{ij} are close to zero. (b) Histogram of the positive correlation coefficients.

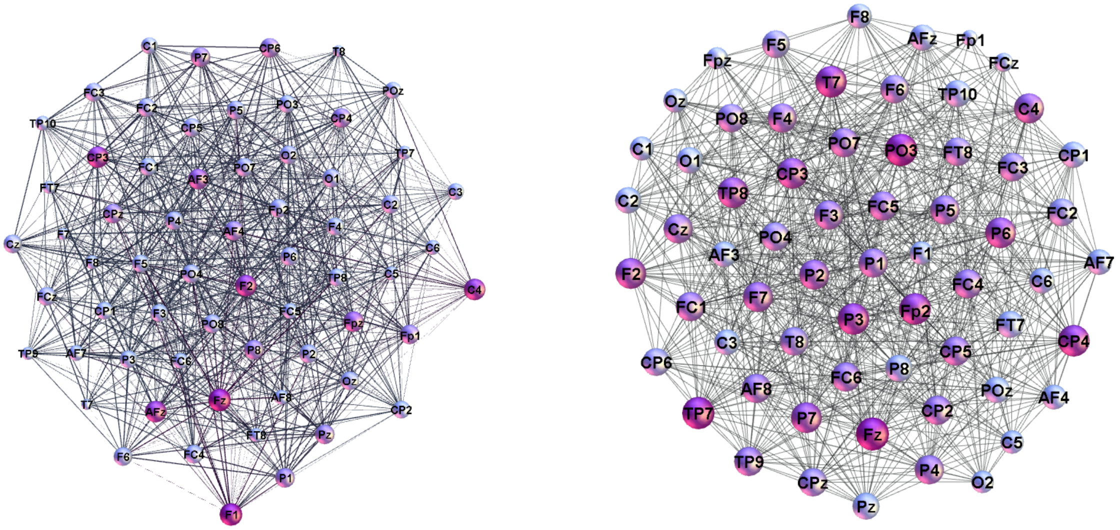

Fig B: An example of the randomised SBN. The listener's L_{2-3} SBN after the randomisation procedure that preserves the node's degree (Random-K) and the total number of links (Random-L), left to right.

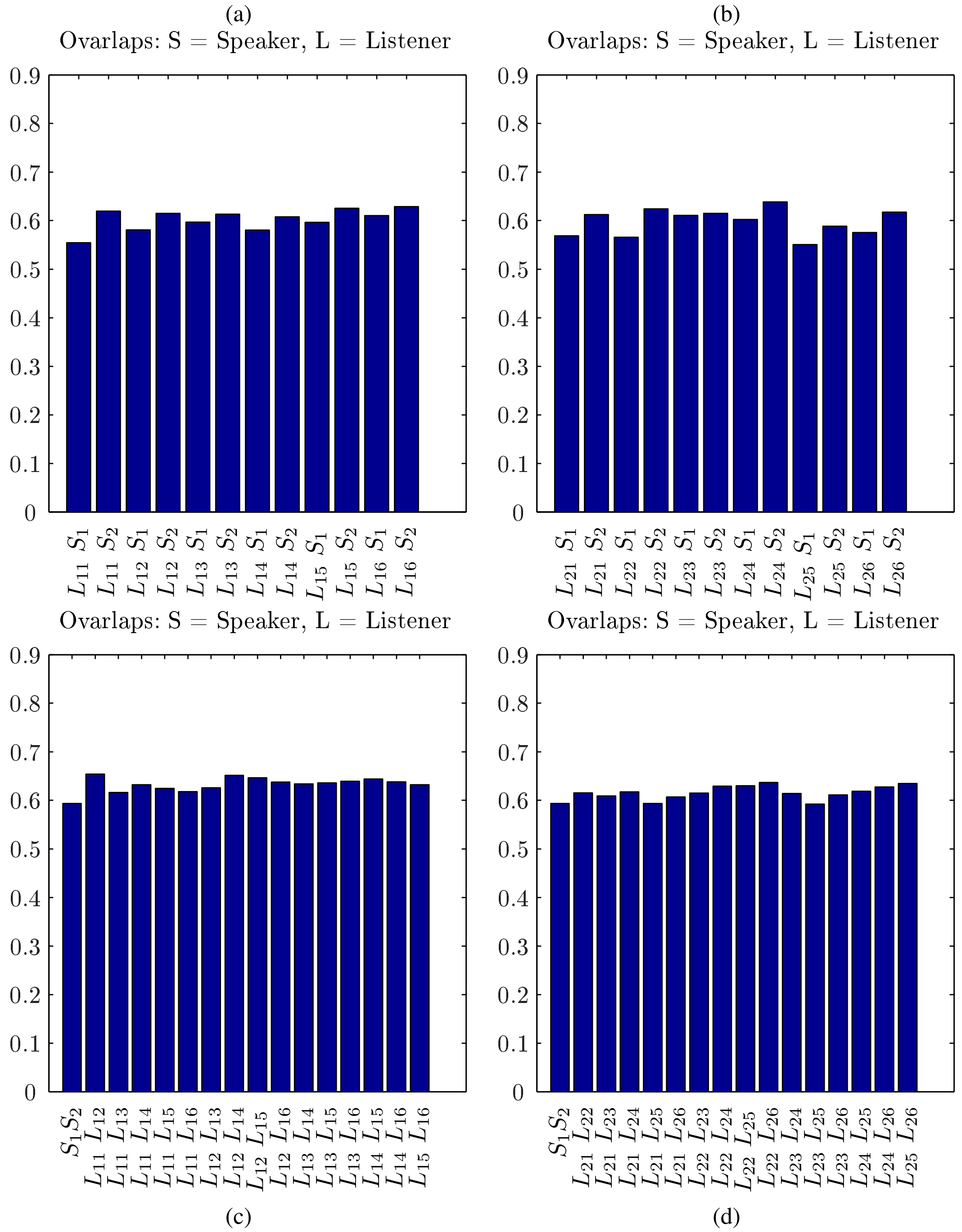

Fig C: Overlaps of the links in the single-brain networks randomized such to preserve the node's degree (Random-K) in stimulus1. The mean $< O_{RK} >$ and the standard deviation σ_{RK} are (0.6024,0.0218), (0.5974,0.0272),(0.6327,0.0150), and (0.6153,0.0142) in the graph (a),(b),(c), and (d), respectively. For a better comparison, the overlaps are plotted on the same scale as the ones on Fig.6 in the paper.

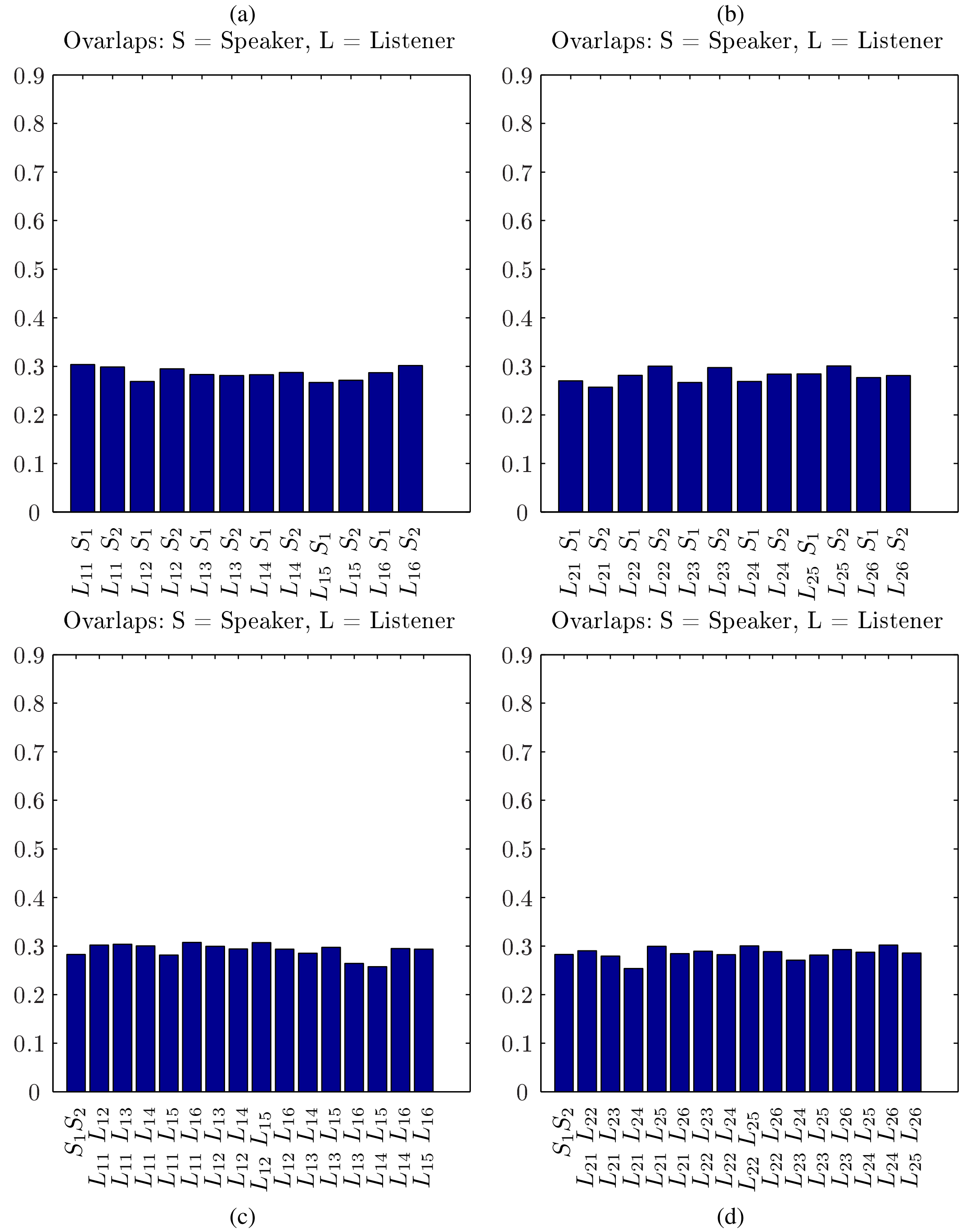

Fig D: Overlaps of the links in fully randomized (Random-L) single-brain networks with the same number of links in stimulus1. The mean $< O_{RL} >$ and the standard deviation σ_{RL} are (0.2855, 0.0125), (0.2806,0.0138), (0.2914,0.0143), and (0.2855,0.0118) in the graph (a),(b),(c), and (d), respectively.

In both randomized models, the overlaps in S3 Fig and S4 Fig are significantly lower than the corresponding overlaps in the original SBNs, cf. Fig 6 in the paper, where the corresponding values $< O >$ and σ are: (0.7666,0.0581), (0.7403,0.0583), (0.6917,0.0796),and (0.7016,0.0723), respectively. In the case of stimulus11 the corresponding values are: (0.7382,0.0569), (0.7222,0.0679), (0.7064,0.0421) and (0.6803,0.0527).
